# Single-cell transcriptomics of peripheral blood mononuclear cells indicates impaired immune and inflammatory responses in alcohol-associated hepatitis

**DOI:** 10.1016/j.humimm.2023.110735

**Published:** 2023-11-30

**Authors:** Xiaochen Liu, Zhang-Xu Liu, Timothy R. Morgan, Trina M. Norden-Krichmar

**Affiliations:** a Department of Epidemiology and Biostatistics, University of California, Irvine, CA, USA; b Division of Gastrointestinal and Liver Diseases, Department of Medicine, University of Southern California, Los Angeles, CA, USA; c Medicine and Research Services, VA Long Beach Healthcare System, Long Beach, CA, USA; d Department of Medicine, University of California, Irvine, CA, USA

**Keywords:** Single-cell RNA-seq, Alcohol-associated hepatitis, Inflammatory cytokines, Immune response, PBMC

## Abstract

Alcohol-associated hepatitis (AH) is often diagnosed at advanced stages, and severe AH usually carries poor prognosis and high short-term mortality. In addition, it is challenging to understand the molecular mechanisms of immune dysregulation and inflammation in AH due to the cellular complexity and heterogeneity. Using single-cell RNA sequencing, previous studies found that AH causes dysfunctional innate immune response in monocytes, involving activation of pattern recognition receptors (PRRs) and cytokine signaling pathways. To better understand the coordinated systemic immune response in AH patients, we performed combined single-cell transcriptome, cell-surface protein, and lymphocyte antigen receptor analysis of peripheral blood mononuclear cell (PBMC) samples. Our results showed inflammatory cytokines and chemokines were highly expressed in AH, including IL-2, IL-32, CXC3R1 and CXCL16 in monocytes and NK cells, whereas HLA-DR genes were reduced in monocytes. In addition, we also found altered differentiation of T-helper cells (T_H_1 and T_H_17), which could further lead to neutrophil recruitment and macrophage activation. Lastly, our results also suggest impaired NK-cell activation and differentiation in AH with reduced gene expression of KLRC2 and increased gene expression of KLRG1. Our findings indicate different mechanisms may be involved in impaired immune and inflammatory responses for the cellular subtypes of the PBMCs in AH.

## 1. Introduction

Long-term heavy drinking can result in alcohol-associated hepatitis (AH), which is characterized by hepatocellular injury (e.g., necrosis), acute hepatic inflammation, and liver fibrosis [1]. With a rapid fibrosis progression rate of 10–20 % per year, more than 70 % of patients with AH will eventually develop cirrhosis [2–4]. AH is often diagnosed at a very advanced stage, and severe AH carries a poor prognosis and high short-term mortality [1,4]. It is generally accepted that the initial etiology of AH pathogenesis is direct toxic effects of alcohol metabolites on hepatocytes and subsequent inflammation leading to hepatocyte death and decreased regeneration of hepatocytes [1,4], as well as increased collagen synthesis, leading to cirrhosis [5–9]. However, it is challenging to understand the molecular mechanisms of immune dysregulation and inflammation in AH due to the complexity and heterogeneity of the immune cells involved.

Single-cell RNA sequencing (scRNAseq) is a recently developed technology that has a greater capability than traditional bulk RNA sequencing to assess transcriptomics into a finer resolution, and thereby resolve cellular complexity and heterogeneity [10]. While most studies related to liver disease which used peripheral blood mononuclear cells (PBMCs) explored scRNAseq profiles in advanced liver diseases, such as cirrhosis and hepatocellular carcinoma [8,11], Kim et al. investigated the complex network of peripheral blood immune profiles in AH, by performing a lipopolysaccharide (LPS) challenge which is relevant to alcohol consumption in humans [12]. Their study found that monocytes expressed functionally diverse responses. In comparing the monocytes from AH patients to those from healthy controls, monocyte markers CD14, C-type lectin receptors (CTRs) MDL, and complement receptor C5AR1 were upregulated, whereas CD16 was down-regulated. Such findings indicated dysfunctional innate immune responses. Another study provided a closer look at the diversity of immune responses [13], using experimental simulations of chronic heavy drinking (CHD) in non-human primates, while integrating transcriptomics, epigenetics and surface antibodies to examine a more complete alcohol-induced immune response [13]. They not only found consistent expression patterns of CD14 and CD16 as mentioned above, but also found that accumulation of monocytes expressing hypoxia (HIF1A) and NFkB signaling pathway were observed in CHD primates.

Prior to the availability of scRNAseq and the development of suitable analysis procedures, many studies lacked clearly identified cell populations and comprehensive evaluations of cellular heterogeneity. In addition, more human studies were needed to thoroughly investigate peripheral immune cell populations on AH. Therefore, to better understand the coordinated systemic immune response in AH patients, we performed combined single-cell transcriptome, cell-surface protein assessment, and lymphocyte antigen receptor (TCR and BCR) analysis of peripheral blood mononuclear cell samples from participants. By combining evidence from cell-surface protein and T-cell and B-cell receptor repertoire, we were able to examine peripheral immunity in a deeper and more comprehensive fashion.

## 2. Methods

### 2.1. Study population and sample collection

Blood samples from an IRB-approved study by the Southern California Alcoholic Hepatitis Consortium (SCAHC) were collected at baseline with consent by the participants from alcohol-associated hepatitis (AH, n = 3) and healthy controls (CT, n = 2). Peripheral blood mononuclear cells (PBMC) were freshly isolated from the blood samples by Ficoll-Histopaque (GE Healthcare) gradient centrifugation. The samples are stored in culture media containing DMSO/FBS according to the protocol specific for 10x Genomics scRNAseq (CG00039), flash frozen, and then stored in a liquid nitrogen tank.

### 2.2. RNA-seq library and V(D)J library construction

Samples were sequenced in 2 batches. For Batch 1 (3′ scRNAseq), the scRNA-seq library preparation was performed according to the Chromium Single Cell 3′ Reagents kit v2 user guide (CG00052). For batch 2 (5′ scRNAseq, cell surface protein (CSP) and V(D)J libraries), cryopreserved cells were prepared for use in 10x Genomics (10x Genomics, Pleasanton CA) single cell protocols according to the demonstrated workflow for flash frozen human peripheral blood mononuclear cells for single cell RNA-sequencing (CG000208).

### 2.3. Sequencing reads alignment and quality control

**Fig. 1.**
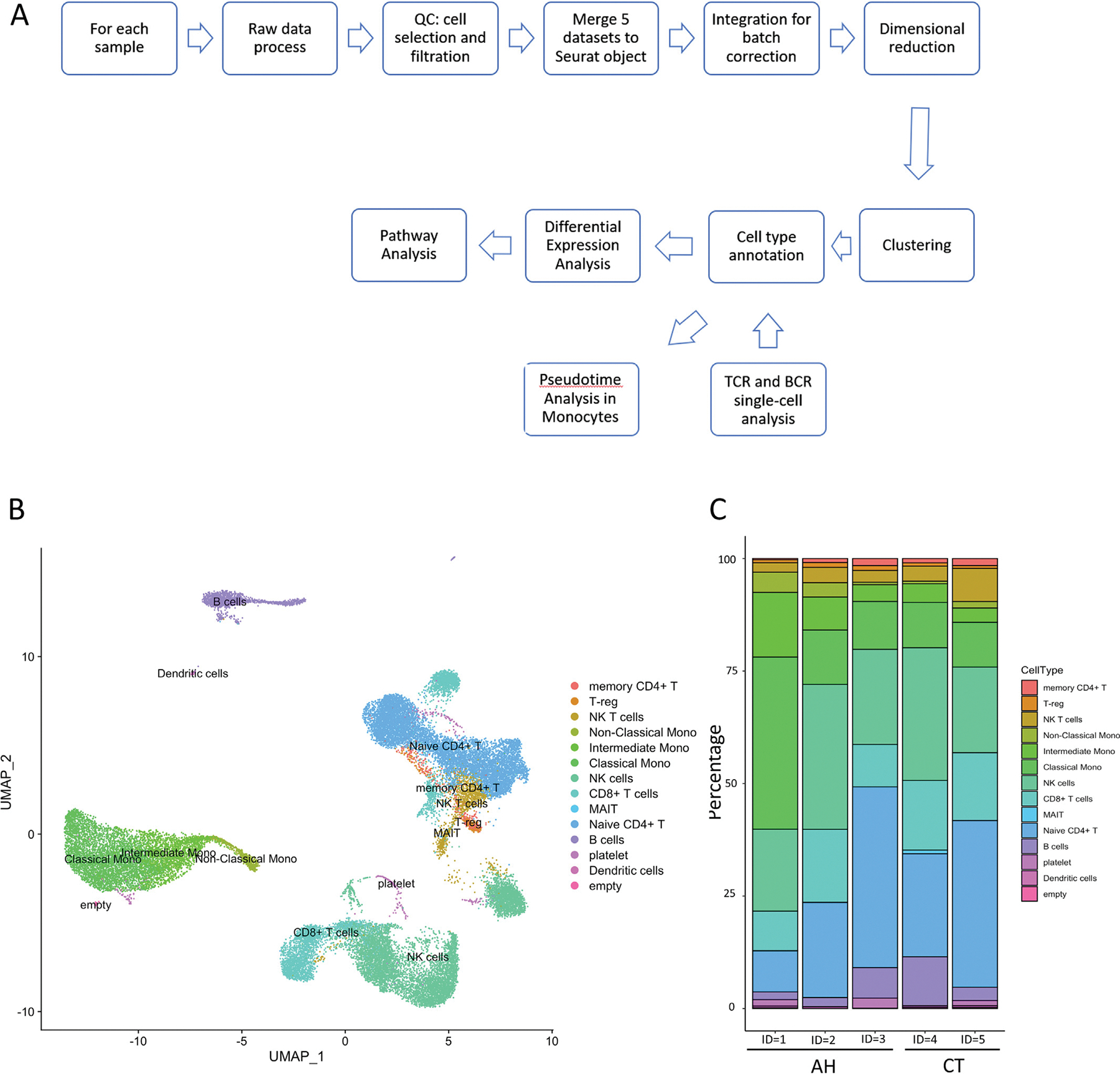
Data analysis flowchart and cell type annotation. A) Analysis flowchart for single-cell data. B) UMAP plot of cell clusters from integrated scRNA-seq samples colored by cell types. C) Histogram of cell proportion from each participant, colored by cell types.

The data analysis flowchart is shown in Fig. 1A. Sequencing reads from gene expression, cell surface protein (CSP) and V(D)J libraries were processed by 10x Genomics Cell Ranger 6.0.2 [10]. Specifically, gene expression and V(D)J data were aligned to the human GRCh38 reference genome, and alignment for CSP data was referenced by the barcode sequences provided. Cell calling was performed using the *cellranger multi* function as recommended by the *cellranger* tutorial. For each sample, we adapted an interquartile range (IQR) based filtering method [14] to remove low quality cells. In addition, genes that were expressed in fewer than 3 cells were removed from the counts matrix. The R package Seurat (version 4.1.0) [15,16] was applied to perform integration and batch correction, dimensional reduction, clustering, and differential expression analysis.

Additional details about the library preparation protocol, sequencing, and quality control are available in the supplemental files.

### 2.4. Cell type annotation

Cell types were annotated using a combination of manual and automated reference-based methods [17–19]. For the manual annotation method, canonical cell markers were chosen based on prior biological knowledge in the literature and in Seurat, and from a manually curated platform of cell markers in human and mouse *CellMarker* [20]. We first used gene expression markers, and then validated with cell surface protein markers (CSP), if available for that cell type. For the automated annotation using *SingleR* [21], Novershtern hematopoietic dataset was used as the reference data because its cell type pool was the most closely matched to our dataset, and it outperformed other reference datasets. The mucosal-associated invariant T (MAIT) cell and invariant natural killer T (iNKT) cells were identified by T cell receptor V(D)J data. Specifically, MAIT cells express gene segments TRAV1–2(Vα7.2)- TRAJ33 (Jα33); iNKT cells express TRAV10 (Vα24)- TRAJ18 (Jα18). Due to limited cell counts, iNKT cells were combined with the NKT cells for downstream analyses. There were no gamma delta chains detected in our TCR data, thus we were not able to identify any gamma-delta T cells. B cell receptor V(D)J data was used to validate the B cell cluster annotation.

### 2.5. Differential expression analysis and pathway analysis

To examine the differences in the gene expression and biological pathways associated with AH compared to healthy control, we first excluded mitochondrial genes and ribosomal genes. Then, for each cell type, differentially expressed genes (DEGs) between healthy controls and AH patients were filtered to retain those with fold change ≥ 1.5 with adjusted P-value <=0.05. The DEGs were also filtered to retain those with detection frequency (pct.1 and pct.2) ≥ 10 % in both two condition groups, and ≥ 25 % frequency in at least one condition groups, to mitigate technical noise. Finally, to examine differences with respect to the number of cells and expression level from each cell, only genes whose difference in detection frequency between the two groups was bigger than 15 % were retained. Batch effects due to the two different platforms (3′ and 5′) were adjusted using logistic regression for all cell types except for MAIT cells. For MAIT cells, since they were only identified using TCR data from batch 2, there was no need to correct for batch effects. Also, since the cell count was very low, the Poisson test was performed for the MAIT cells to find DEGs with ≥ 1.5-fold change and adjusted P-value < 0.01.

We then performed KEGG pathway enrichment analysis using *clusterProfiler* [22]. Specifically, enriched pathways were calculated by cell-type clusters and directionality of DEGs.


Genes=cell-typecluster+direction

Cell-type clusters were combined into the following 4 groups: (1) B cell group, (2) T cell group, (3) NK and NKT group, and (4) monocytes and dendritic cell group.

### 2.6. Pseudotime analysis on monocytes

Previous studies have highlighted the considerable alterations induced by alcohol-associated liver diseases in myeloid cells, especially in monocytes [13,23–26]. Monocytes are leukocytes that circulate in the blood, and can differentiate into macrophages. Monocytes are commonly categorized into three sub-types: classical (CM), intermediate (IM) and non-classical (NCM). Each sub-type has unique functions involved in chronic inflammation [27]. To further understand the mechanism and dynamic changes of the 3 subsets of monocytes, we performed statistical pseudotime estimation, to attempt to reconstruct the relative temporal ordering of the cells based on similarities in their expression patterns, which is referred to as “pseudotime analysis” [28]. The pseudotime was constructed by *slingshot* [29], followed by differential expression analysis along trajectories by *tradeseq* [30]. Lineages were constructed using the PCA reduced dimension as input. To investigate expression patterns associated with the disease condition and trajectory, we fitted a negative binomial generalized additive model (NB-GAM in *slingshot*) for each gene, allowing 6 knots for every lineage. As recommended as the default settings for slingshot, significant genes were those with ≥ 2-fold change and FDR-corrected P-value ≤ 0.05. Additional details are available in the supplemental files.

All downstream analyses, after running the *cellranger* software, were performed using R software version 4.1.3.

## 3. Results

### 3.1. Demographic, clinical, and sequencing data

The demographic, clinical and sequencing information of the 5 Hispanic participants is listed in Table 1. All 3 AH patients were male, with age ranges of 38–53 years, and with BMI values in the overweight or obese ranges. Also, their AH conditions were severe based on liver disease scoring metrics: Maddrey’s discriminant function (DF) score (range: 48–61), MELD (range: 23–28), and Childs-Pugh (range: 11–14). The two healthy controls were also overweight, and both had liver disease severity scores in the healthy range (DF = 0, MELD = 7 and Child-Pugh = 5). The gene expression (GEX) data was determined from the samples, which each had cell counts between 6,000 cells to 10 k cells per sample. There were less cells called from the cell surface protein data (CSP) than GEX. The V(D)J-T cell counts varied for each participant, with ranges of 1491–4017 cells. There were fewer V(D)J-B cells (156 cells and 329 cells) in AH than control (1028 cells).

### 3.2. Differences in cell populations between AH and CT

In total, we analyzed sequencing data for 40,270 cells for gene expression analysis (Table 1). 32,848 cells passed the quality control steps. Following integration, dimensional reduction, and clustering, we obtained 26 clusters. Thirteen cell types were identified using a combination of manual and automated method stated in the method section, and further validated using available CSP markers (Fig. 1, Suppl. Figs. 1–4, Suppl. Table 1). TCR and BCR data were also used to confirm our annotation. In addition, using TCR data we identified 67 MAIT cells and 10 iNKT cells (the iNKT cells were combined with NKT cell cluster). In the AH samples, we found higher percentages among all 3 types of monocytes, with an average of 2-fold increase in cell counts (Fig. 1C, Suppl. Table 1). However, NKT cells and MAIT cells had lower abundance in AH compared to healthy controls.

### 3.3. Differences in gene expression and pathways

**Fig. 2.**
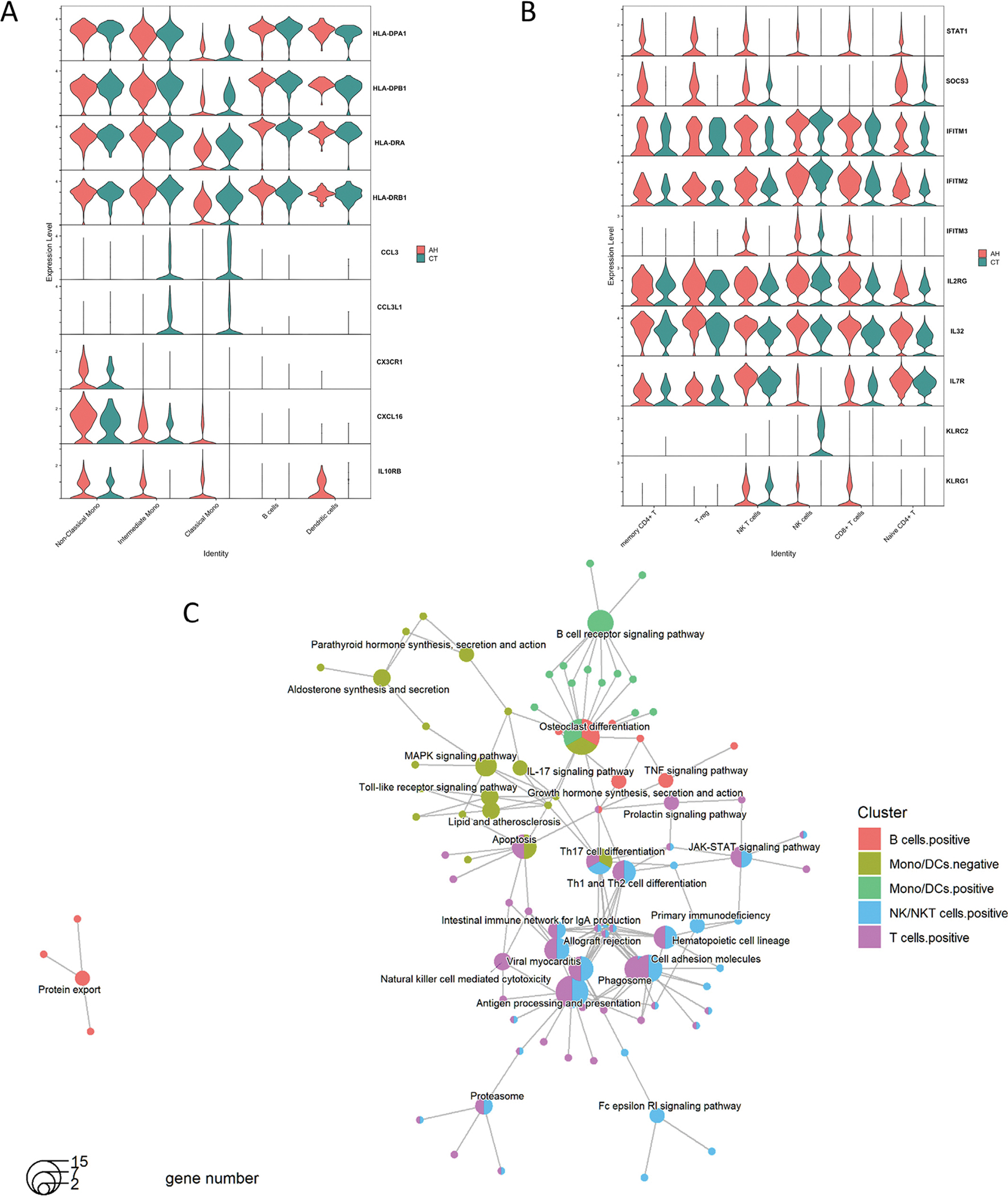
Expression levels of select genes and KEGG enrichment analysis. Expression levels of select genes from A) monocytes, B cells and dendritic cells; and B) T cells, NKT cells and NK cells shown as violin plot. Red represents AH samples and green represents healthy control. C) Network visualization of KEGG enrichment analysis of top 308 DEGs by cell type similarity and regulation directionality of gene expression. Cell types were categorized to 4 groups: group 1 = CD4, CD8; group 2 = The 3 types of monocytes + DCs; group 3 = NK, NKT; group 4 = B cells. (For interpretation of the references to colour in this figure legend, the reader is referred to the web version of this article.)

In comparison to the control samples, differentially expressed genes (DEGs) from each immune cell population exhibited several impaired innate and adaptive immune responses via diverse pathways in AH. First, chemokine genes CCL3 and CCL3L1, which are associated with toll-like receptor signaling pathway, had much lower expression levels in AH monocytes, compared to CT monocytes (Fig. 2A and C, Suppl. Figure 5A, Suppl. Figure 6). Additionally, AH monocytes also demonstrated defects in antigen presenting and MAPK pathway with reduced gene expression levels of HLA-DR genes (MHC class II), and upregulation of anti-inflammatory cytokine IL-10. However, AH samples also had higher expression of leukocyte immunoglobulin-like receptor genes (LILRs) from monocytes/DC cells that are related to B cell receptor signaling.

In addition to monocytes, cytotoxic and T helper cells also demonstrated differences in gene expressions and pathways between AH and CT. Genes over-expressed in AH NK cells, NKT cells, CD4 + T cells and CD8 + T cells, were associated with various cytotoxic activities. Cytokine signaling gene STAT1 was highly expressed in NKT, CD8 + T and naïve CD4 + T cells, and SOCS3 was highly expressed in regulatory T and naïve CD4 + T (Fig. 2B and C, Suppl. Figure 5B, Suppl. Figure 7). Pathway analysis confirmed the upregulation of Th cell differentiation and the JAK-STAT signaling pathway. NKT cells in AH also showed high expression of Interferon-Induced Transmembrane Protein IFITM2 and IFITM3 genes which are markers of phagosome and apoptosis. The activating NK-cell receptor KLRC2 gene expression in NK cells was decreased greater than 3-fold in AH compared to CT (log_2_FC = 1.8, P_adj_ = 2 × 10^−306^, Suppl. Figure 8). The expression of the ITIM-Containing Receptor KLRG1, which is an inhibitory receptor related to inhibition of NK cell activation and cytotoxic activity, was increased in NK and CD8 + T cells in AH. In addition, proinflammatory cytokine genes, IL2RG and IL32, as well as cytokine-cytokine receptor interaction marker, IL7R, were significantly upregulated in NK cells from AH samples. These cytokine genes are associated with TNF-alpha signaling via NF-kB pathway. Lastly, B cells from AH samples exhibited the highly expressed genes FOS, FOSB and JUNB, which are related to TNF signaling and osteoclast differentiation pathways (Fig. 2C, Suppl. Figure 9).

### 3.4. Heterogeneity and dynamic changes in monocytes

**Fig. 3.**
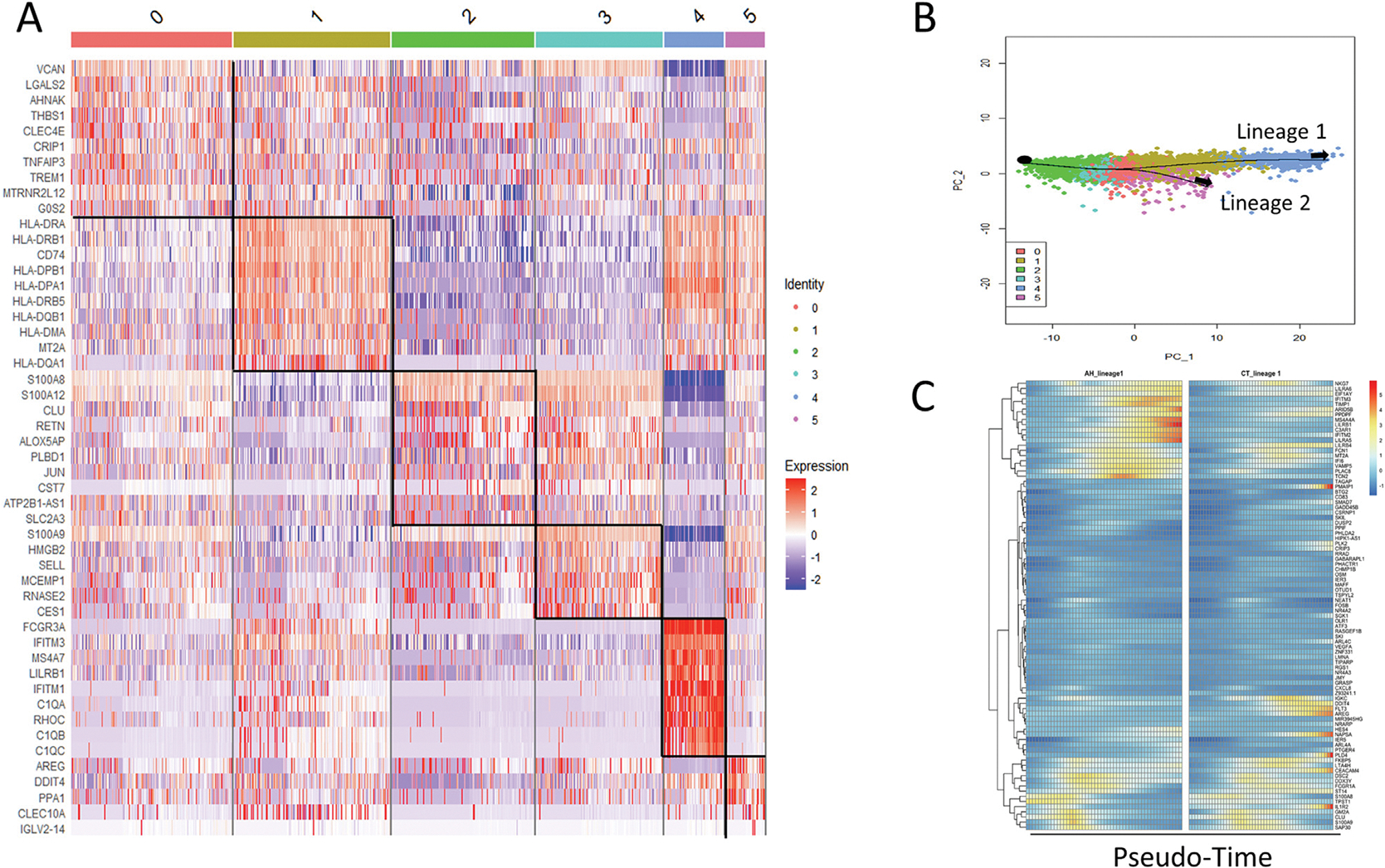
Pseudotime analysis of monocytes. A) Heatmap of most variable genes in monocytes grouped by clusters. B) Pseudotime analysis of monocytes determined by Slingshot. C) Heatmap of expression levels of top 87 genes that most expressed differently across pseudotime and across conditions by lineage 1 (full lineage, goes from CM (C2, C3, C0) to IM(C1) then to NCM(C4)). X-axis represents pseudotime from left to right.

To further understand the differential expression patterns over monocyte states and their association with AH, we performed a pseudotime analysis. Monocytes were re-clustered into 6 clusters, which were further annotated using canonical markers (Fig. 3A). Cluster 0 (C0), Cluster 2 (C2), and Cluster 3 (C3) were categorized as CD14 + and CD16-, which were identified as classical monocytes (CM). Cluster 1 (C1) and Cluster 5 (C5) highly expressed HLA-DR genes, suggesting that they were intermediate monocytes (IM). Cluster 4 (C4) was the only cluster that showed a high expression level of CD16 and low expression of CD14, and was therefore categorized as non-classical monocytes (NCM).

Two lineages were constructed beginning with state C2 (Fig. 3B). Lineage 1 progressed from CM (C2, C3, C0) to IM(C1) then to NCM (C4). Lineage 2 progressed from CM (C2, C3, C0) to IM(C5). Since lineage 1 covered all 3 states of monocytes, we evaluated changes in gene expression as a function of pseudotime within lineage 1. We identified the 87 most diverse genes as those that had the most significant changes in expression across pseudotime and between AH and CT (Fig. 3C, Suppl. Figure 10, Suppl. Table 4).

These temporal and conditionally diverse genes are related to immune responses and oxidative stress (Suppl. Figure 11), including TNF-alpha signaling via NF-kB (associated genes PTGER4, DUSP2, BTG2,CD83, GADD45B, TIPARP, PLK2), p53 pathway (associated genes TSPYL2, BTG2, ST14, TCN2), apoptosis (associated genes IFITM3, BTG2, GADD45B, LMNA), hypoxia (associated genes PLAC8, MT2A, TIPARP, MAFF) and inflammatory response (associated genes PTGER4, BTG2, CXCL8, RGS1, OSM).

To investigate the overall expression patterns in monocytes, we also examined the differential expression patterns across pseudotime within each lineage (Suppl. Figure 12). AH showed higher expression than CT throughout pseudotime. Average expression level was steadily increasing in the first half of pseudotime, then showed exponential increase of expression level in the later stage in lineage 1(CM-IM-NCM), while in lineage 2 (CM-IM) the average expression level was relatively steady across states, with an increasing trend at later IM stage.

## 4. Discussion

Heavy alcohol consumption has been proven to severely damage liver cells, leading to a broad spectrum of liver diseases, including alcohol-associated hepatitis (AH). One of the potential contributors to high mortality in AH may be a defective immune defense [31]. However, the altered alcohol-induced immune response in PBMCs is not entirely understood, partially due to its high cellular heterogeneity and the complex nature of immunity. ScRNA-seq has the ability to resolve cellular complexity and dissect complicated immune responses of PBMCs of AH. However, because of this emerging new technology, high costs and strict tissue requirements, available studies are limited and there is a lack of data from diverse ethnicities [13,32]. This study evaluates the PBMC immune profiles in participants with AH, by combining single-cell transcriptomics, CSP and V(D)J data for BCR and TCR. We identified 13 types of cells and found differences in both cell composition and gene expression associated with AH. We further discovered altered innate and adaptive immune responses.

Circulating monocytes can migrate to sites of inflammation and injured tissue and differentiate into macrophages in the tissue. Both blood monocytes and macrophages/Kupfer cells have been implicated in hepatic injury, inflammation, regeneration and fibrosis in ALD and AH. Our results confirmed the heterogeneity of monocytes by identifying three subsets of monocytes with 6 sub-clusters: classical, intermediate, and non-classical monocyte. Interestingly, this high heterogeneity in monocytes was also observed in alcohol-fed non-human primates, where they identified 9 monocyte subsets by scRNA-seq analysis [13,23]. In terms of disease-related features, in addition to up-regulated expression of pro-inflammatory cytokine and chemokine genes, we observed an average of 2-fold increase in cell percentages across all subtypes of monocytes in AH compared to CT, indicating a pro-inflammation state in AH. However, we also observed decreased gene expression levels in HLA-DR genes (MHC class II), and upregulation of IL-10 in AH [12,25]. This consistent evidence suggests that chronic alcohol drinking may lead to down-regulation of genes with immune and anti-viral responses, and defects in presenting antigen to T-cells in the monocytes. Similar to the study with alcohol-fed non-human primates, we also found up-regulated cytokine CXCL16 and lysosomal protein CD63, but we did not replicate DEGS of TLR8, CD86, IL6 and IL-8 found in these studies [13,23].

**Fig. 4.**
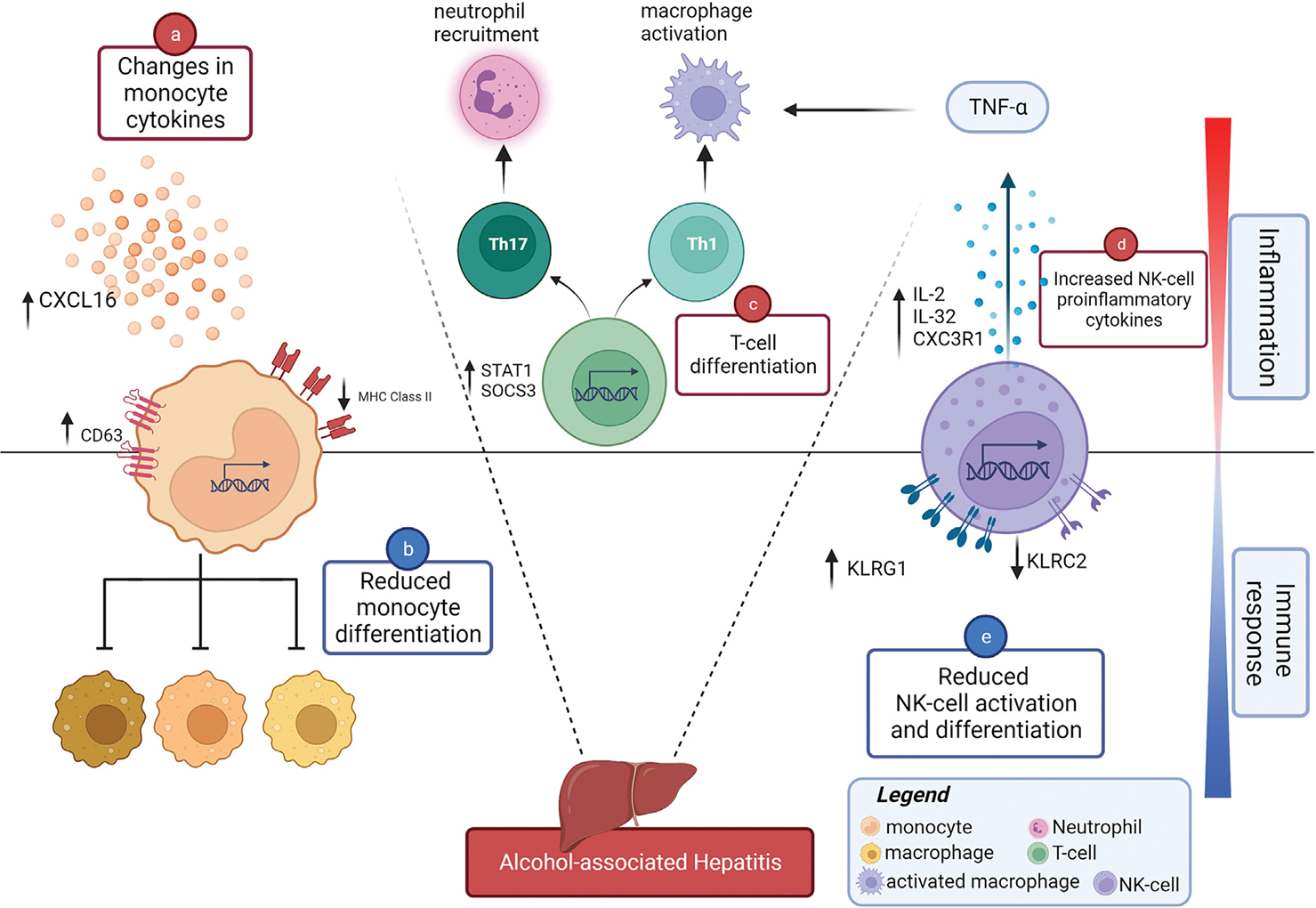
Mechanism illustration of the inflammation and immune responses in AH. Illustration of the inflammation and immune responses in peripheral T-cells, NK cells, and monocytes from AH patients. Created with BioRender.com.

Additionally, pseudotime analysis found 2 lineages that differentiated to 3 correlated monocytes subtypes. Genes were differentially expressed across pseudotime depending on disease condition [33], and such dynamic and global changes were observed in both lineages (Fig. 3 and Suppl. Figure 12). The most significant DEGs were associated with TNFα signaling via NF-kB pathway [34–36], inflammatory response [12,37], apoptosis and hypoxia pathway [38], which suggest impaired functions of monocytes in AH [39,40]. Together, our results demonstrated complex altered monocyte responses in AH, suggesting a mixed population of both inflammatory and anti-inflammatory or repair-promoting monocytes in AH (Fig. 4). This might reflect dynamic functional and phenotypical changes of monocytes during the chronic process of injury, inflammation, regeneration and fibrogenesis in the liver of AH patients. Increased numbers of monocytes and up-regulation of pro-inflammatory genes in monocytes could promote acute hyper-inflammation in AH. Monocytes are antigen-presenting cells that load antigen on MHC class I and II molecules and prime CD8^+^ and CD4^+^ T cells [41]. However, defects of monocytes in antigen presentation in AH reflected by down-regulation of HLA-DR genes and up-regulation of anti-inflammatory cytokine IL-10 may contribute to dysfunctional immune responses to secondary viral and bacterial infections frequently observed in AH patients.

In addition to a decreased response of secondary infections by monocytes, multiple studies have reported a low frequency of NK cells in AH and ALD patients [42,43], indicating a defect of NK cells. In this study, we found evidence suggesting impaired immune responses in NK cells in AH (Fig. 4). Our results demonstrated decreased expression of activating NK-cell receptor KLRC2, and increased expression of ITIM-containing inhibitory receptor KLRG1, in NK cells in AH. This may suggest that the imbalance of activating and inhibiting signaling pathways on NK cells could lead to inhibition of NK cell activation and proliferation, thus impairing NK cell function in AH. This impairment of NK cell function, in turn, may be associated with increased susceptibility to secondary infections in AH patients. Despite the decreasing trend of circulating NK cells, we found an increase of cytotoxicity activities by NK cells. Over-expression of IL2 increases the expression of IL32 [44,45]. IL-32 induces the production of TNFα in macrophages [46], suggesting that NK cells may play a role in promoting TNFα signaling pathway. Lastly, we also observed the proinflammatory chemokine receptor CX3CR1 gene was expressed higher in AH than in CT, which is consistent with previous findings in severe AH patients [31]. The CX3CR1 gene is involved in endothelial progenitor cells (EPCs) apoptosis by NK cells, which could alter endothelial repair and tissue regeneration.

Proinflammatory cytokines were highly expressed in both naïve CD4+, memory CD4+, and CD8 + T cells, which may further promote chronic inflammation in AH patients. In addition, differentiation to T_H_1 cells was an enriched pathway in T cells due to upregulated genes, including STAT1 and SOCS3 [47,48], and T_H_1 cells further help activate macrophages and lead to tissue inflammation. In addition to Th1 cells, another proinflammatory-related cell type,T_H_17 cell differentiation, also was found to be enriched in AH [49]. T_H_17 cells enhance neutrophil response and have been implicated in hepatic inflammation of AH [50]. Taken together, activation of this proinflammatory cascade and the resulting neutrophil recruitment [51], may lead to aggravation of inflammation and injury in the liver [52].

Additionally, due to the enhanced capabilities of multi-omics single-cell sequencing technology, we were able to identify some rare cell types in our samples, such as NKT cells and MAIT cells. NKT cells display features of both innate and adaptive immunity, and are characterized by expressing both T-(TCR) cell and NK cells markers [53]. There is limited information about the functions of peripheral NKT cells in regards to alcohol-associated liver diseases, possibly due to the rapid downregulation of surface markers and/or apoptosis of activated NKT cells [54]. Our study identified type I and type II NKT cells, with CT participants having almost double the cell counts as the AH participants. This may imply impaired function of NKT cells in AH. Surprisingly, Interferon-Induced Transmembrane Protein IFITM2 and IFITM3 genes were highly expressed in AH in NKT cells, suggesting that NKT cells are activated with less frequency, which may be due to activation-induced cell death [55]. MAIT cells, a subset of innate-like T cells, were also found to be significantly deficient in AH patients [53,56]. This may indicate dysfunction of MAIT cells, which may lead to further increased risk of secondary bacterial infection in AH [57,58].

There are several potential limitations of this study. First, because it was a preliminary study, the sample size is small, 3 AH and 2 healthy controls, which could limit the generalizability of the results. However, we made best efforts to closely match the BMI in the AH and CT participants, and disease severity in all AH participants, in order to reduce heterogeneity within the disease vs. control conditions. Second, the samples were processed in two batches, using different protocols (3′ scRNAseq vs. 5′ scRNAseq with V(D)J profiling and cell surface protein markers). Nevertheless, we were able to batch correct the samples to provide an integrated analysis which gave us stronger confidence in our results. Specifically, we applied and evaluated multiple batch correction methods, including Seurat Multi CCA (canonical correlation analysis) [16], Seurat SCTransform [59], and Harmony [60], to obtain the most robust results for downstream analyses (Suppl. Figure 1). Third, the study evaluated only Hispanic participants to eliminate confounding issues that may be introduced by ethnicity. While it is a strength of our study to be among the first to use scRNA-seq technology with AH PBMC samples from a Hispanic population, we acknowledge that future studies will be necessary to evaluate PBMC scRNA expression in other ethnicities with larger sample size.

In summary, this study presented an in-depth analysis of several cell surface markers and single cell RNA expression among peripheral PBMCs in alcohol-associated hepatitis in a small number of participants. Our findings indicated several mechanisms of impaired immune and inflammatory responses for different cell types in AH. We also presented an analysis pipeline for small sample size with multiple platforms for scRNA-seq analysis. Future studies focusing on prognostic and predictive biomarkers in liquid biopsy with larger sample sizes are necessary.

## Supplementary Material

Supplementary Table

Supplementary Material

Appendix A. Supplementary material

Supplementary data to this article can be found online at https://doi.org/10.1016/j.humimm.2023.110735.

## Figures and Tables

**Table 1:** Demographic, clinical, and sequencing parameters for each subject.

Study ID	Demographics			Clinical Parameters						Sequencing (n = 40270 cells)			
			
	Diagnosis	Sex	Age (year)	BMI	DF score	MELD	Childs Pugh	AST (U/L)	ALT (U/L)	GEX (n = 40,270 cells)	VDJ-T (n = 8723 cells)	VDJ-B (n = 1513 cells)	CSP (n = 24,233 cells)

1	AH	Male	48	27	58	28	12	162	63	6647	1491	156	6757
2	AH	Male	53	37	61	23	14	91	50	8493	4017	329	8796
3- (batch1)	AH	Male	38	25	48	24	11	124	36	10,012	–	–	–
4	Healthy	Male	19	31	0	7	5	18	12	8598	3215	1028	8680
5- (batch1)	Healthy	Female	45	30	0	7	5	18	13	6520	–	–	–

## Abbreviations:

AH: Alcohol-associated hepatitis
BCR: B-cell receptor
CM: classical monocyte
CSP: cell surface protein
DEG: differentially expressed gene
DF: Maddrey’s Discriminant Function
FDR: false discovery rate
IM: intermediate monocyte
iNKT: invariant natural killer T
ITIM: Immunoreceptor Tyrosine-based Inhibitory Motif
MAIT: mucosal-associated invariant T cell
MELD: model for end-stage liver disease
NCM: non-classical monocyte
scRNAseq: single-cell RNA sequencing
PBMC: peripheral blood mononuclear cell
TCR: T-cell receptor
