# Supplementary material for: Single-cell transcriptomics of peripheral blood mononuclear cells indicates impaired immune and inflammatory responses in alcohol-associated hepatitis
Source: Hum Immunol. Author manuscript; Available in PMC 2025 Oct 23. (PMC12549007; doi:10.1016/j.humimm.2023.110735)
Supplement: Supplementary Material [file NIHMS2115344-supplement-Supplementary_Material.pdf]

SUPPLEMENTAL MATERIALS

TITLE:
Single-cell transcriptomics of peripheral blood mononuclear cells indicates impaired immune and inflammatory responses in alcohol-associated hepatitis

AUTHORS:
Xiaochen Liu[a], Zhang-Xu Liu[b], Timothy R. Morgan[c,d], Trina M. Norden-Krichmar[a*]

AUTHORS' AFFILIATIONS:
[a]Department of Epidemiology and Biostatistics, University of California, Irvine, CA, USA; [b]Division of Gastrointestinal and Liver Diseases, Department of Medicine, University of Southern California, Los Angeles, CA, USA; [c]Medicine and Research Services, VA Long Beach Healthcare System, Long Beach, CA, USA and [d]Department of Medicine, University of California, Irvine, CA, USA

Table of Contents

RNA-seq library and V(D)J library construction using 10x genomics platform 2

Batch 1 (3' scRNAseq) 2

Batch 2 (5' scRNAseq, CSP and V(D)J) 2

Sequencing quality control 3

Pseudotime analysis: Additional results 4

Supplemental Figure Legends 5

Suppl. Figure 1 7

Suppl. Figure 2 8

Suppl. Figure 3 9

Suppl. Figure 4 10

Suppl. Figure 5 11

Suppl. Figure 6 12

Suppl. Figure 7 13

Suppl. Figure 8 14

Suppl. Figure 9 15

Suppl. Figure 10 16

Suppl. Figure 11 17

Suppl. Figure 12 18

References 19

RNA-seq library and V(D)J library construction using 10x genomics platform

Batch 1 (3' scRNAseq)

The scRNA-seq library preparation was performed according to the Chromium Single Cell 3' Reagents kit v2 user guide (CG00052). Briefly, a volume of single cells to target a capture of 10,000 cells was used in the generation of GEMs (gel bead in emulsions). A total of 43.3 µL cell suspension, 40 µL Single Cell 3' Gel Bead and master mix was loaded to the Chip A Single cell to generate single-cell GEMs. Then, the GEMs were transferred to PCR tubes and incubated in a Bio-Rad C1000 Thermocycler (Bio-Rad Laboratories, Hercules, CA) for the reverse transcription protocol. The GEMs were cleaned up using Dynabeads MyOne SILANE (Life Technologies, Carlsbad CA) and then amplified with the cDNA primers using 11 cycles according the 10X workflow. After amplification, the cDNA was cleaned using 0.6X SPRIselect (Beckman Coulter, Indianapolis, IN). Post cDNA amplification reaction quality control and quantification were performed on the Agilent Bioanalyzer 2100 DNA High Sensitivity chip (Agilent). The final Single Cell 3' Libraries contained the P5 and P7 primers used in Illumina bridge amplification PCR. The 10x Barcode and Read 1 (primer site for sequencing read 1) was added to the molecules during the GEM-RT incubation. The P5 primer, Read 2 (primer site for sequencing read 2), Sample Index and P7 primer were added during library construction. The workflow was followed according to the 10X protocol and sequenced on the HiSeq 4000.

Batch 2 (5' scRNAseq, CSP and V(D)J)

Cryopreserved cells were prepared for use in 10x Genomics (10x Genomics, Pleasanton CA) single cell protocols according to the demonstrated workflow for flash frozen human peripheral blood mononuclear cells for single cell RNA-sequencing (CG000208). The washed single cell suspensions were labeled with BioLegend TotalSeq-C TBNK antibodies (BioLegend,

San Diego CA) according to their protocol. Sequencing libraries were prepared using the Single Cell V(D)J v1 protocol from 10X Genomics. Briefly, the 10X workflow was followed using 10,000 cells as the capture target. The resulting Gel-in-Emulsion (GEMs) were transferred to PCR tubes and incubated in a Bio-Rad C1000 Thermocycler (Bio-Rad Laboratories, Hercules, CA) for the reverse transcription protocol. The GEMs were cleaned up using Dynabeads MyOne SILANE (Life Technologies, Carlsbad CA) and then amplified with the sc5' feature cDNA primers using 11 cycles according the 10X workflow. The cDNA was cleaned using 0.6X SPRIselect (Beckman Coulter, Indianapolis, IN) with the supernatant from the first bead separation used for construction of the cell surface protein library. The pellet continued through the protocol and the eluted cDNA used for V(D)J enriched and 5' gene expression libraries. The amplified full-length cDNA was used to enrich full-length V(D)J segments using PCR amplification with primers to both the T and B cells. Amplified full-length cDNA was also used for 5' gene expression library construction. Quality control assays were performed on the cDNA using Qubit DNA HS assay (Life Technologies, Carlsbad CA) and Agilent 2100 Bioanalyzer DNA HS (Agilent, Santa Clara, CA). The cDNAs were fragmented, end repaired and then A-tailed. After a SPRIselect cleanup the adapters were ligated on the cDNA. Sample indexes were added by PCR and a double-sided size selection using SPRIselect was performed. The libraries were assayed for quality using Qubit DNA HS assay, Agilent 2100 Bioanalyzer and quantified by Kapa qPCR Library (Roche, Basel, Switzerland) quantification for Illumina platform. The libraries were sequenced the Illumina NovaSeq 6000 (Illumina, San Diego, CA) using 26 cycles for read 1, 8 cycles for the index read and 100 cycles read 2.

Sequencing quality control

For each sample, we adapted an interquartile range (IQR) based filtering method[1] to remove low quality cells using the equations shown below:

$$upper\ threshold\ (UT) = Q3 + 1.5IQR$$

$$lower\ threshold\ (LT) = Q1 - 1.5IQR$$

where Q1 is the first quantile and Q3 is the third quantile. IQR is the difference between Q1 and Q3. The absolute value was taken if the threshold calculated was negative. Cells that were detected in less than the LT genes and greater than the UT genes, as well as cells that contains more than LT mitochondrial genes were removed from further analysis. In addition, genes that were expressed in fewer than 3 cells were removed from the counts matrix. The R package Seurat (version 4.1.0)[2], [3] was applied to perform integration and batch correction, dimensional reduction, clustering, and differential expression analysis. For each individual sample, data were first log-normalized then scaled to get log-transformed data. Next, highly variable features were identified by *FindVariableFeatures()* with the "vst" method. Then cross-dataset cell-pairs that were in a matched biological state ("anchors") were identified using *FindIntegrationAnchors()* function for data integration to correct for batch effects and further comparative analyses. Principal component analysis was performed using identified variable genes, with the first 30 principal components (PCs) being selected to construct a K-nearest neighbor (KNN) graph based on the Euclidean distance in PCA space. Finally, cells were clustered using modularity optimization method of the Louvain algorithm.

Pseudotime analysis: Additional results

A total of 1339 genes had temporal differences across the monocyte states in CT, 368 genes in AH, and 162 genes expressed differently across pseudotime and across conditions (Suppl. Figure 10). Among these 162 DEGs, we further identified the 87 most diverse genes as those that had the most significant changes in expression across pseudotime and between AH and CT (Figure 3C). Genes such as C3AR1 and IFITM2 were not expressed in the beginning state, but became expressed towards the later state (NCM) in AH condition; whereas in CT, these genes were not

expressed throughout the entire pseudotime states. Some genes, such as PMAIP1, IGKC, DDIT4, FLT3 and AREG, were expressed at relatively low levels across all of the states in AH; but in CT they had no expression in the beginning state, but high expression in later stages.

Supplemental Figure Legends

Suppl. Figure 1: UMAP plot after normalization and integration, colored by individual samples. Each dot indicates one cell, with total of 32,848 cells. Well-mixed clusters showed cells were clustered without impact of batch effects. B, C) UMAP showed V(D)J B-cell receptors (B) and T-cell receptors (C) with background of GEX clustering UMAP.

Suppl. Figure 2: UMAP plot of integrated scRNA-seq dataset colored by cell clusters.

Suppl Figure 3: Panel of 9 cell surface protein (CSP, left) and their associated RNA expression (right) side by side view: CD19, CD3, CD45, CD14, CD16, CD4, CD8, CD56, and CD11c. Color indicates the level of expression.

Suppl. Figure 4: Cell type annotation using manual method (e.g., canonical GEX and CSP markers, left) and SingleR (right). 2 red circles indicate the inconsistent annotations.

Suppl. Figure 5: Expression levels of select genes across the individual samples from A) monocytes, B cells and dendritic cells; and B) T cells, NKT cells and NK cells shown as violin plot. Red represents AH samples and green represents healthy control.

Suppl. Figure 6: Feature plot of CCL3 and CCL3L1 from AH (left panel) and CT (right panel). Each dot represents one cell, and gradient color indicates expression level.

Suppl. Figure 7: Feature plot of STAT1 and SOCS3 from AH (left panel) and CT (right panel). Each dot represents one cell, and gradient color indicates expression level.

Suppl. Figure 8: Feature plot of KLRG1 (ITIM-Containing Receptor) and KLRC2 (activating NK-cell receptor) from AH (left panel) and CT (right panel). Each dot represents one cell, and gradient color indicates expression level.

Suppl. Figure 9: Feature plot of FOS, FOSB and JUNB from AH (left panel) and CT (right panel). Each dot represents one cell, and gradient color indicates expression level.

Suppl. Figure 10: A) Histogram of differentially expressed gene counts from monocytes using pseudotime analysis by condition. B) Heatmap of expression levels of 162 genes (overlapped genes as shown on the left) that expressed differently across pseudotime and across conditions by lineage 1 (full lineage, goes from CM (C2, C3, C0) to IM(C1) then to NCM(C4)). X-axis represents pseudotime from left to right.

Suppl. Figure 11: Barplot of MSigDB hallmark pathway enrichment of top 87 genes sorted by p-value ranking. Red indicates p-values <0.05.

Suppl. Figure 12: Average gene expression levels across pseudotime for each of the lineages from all genes. Green lines are lineage 1, yellow lines are lineage 2. Darker lines are AH samples and lighter lines are healthy control samples.

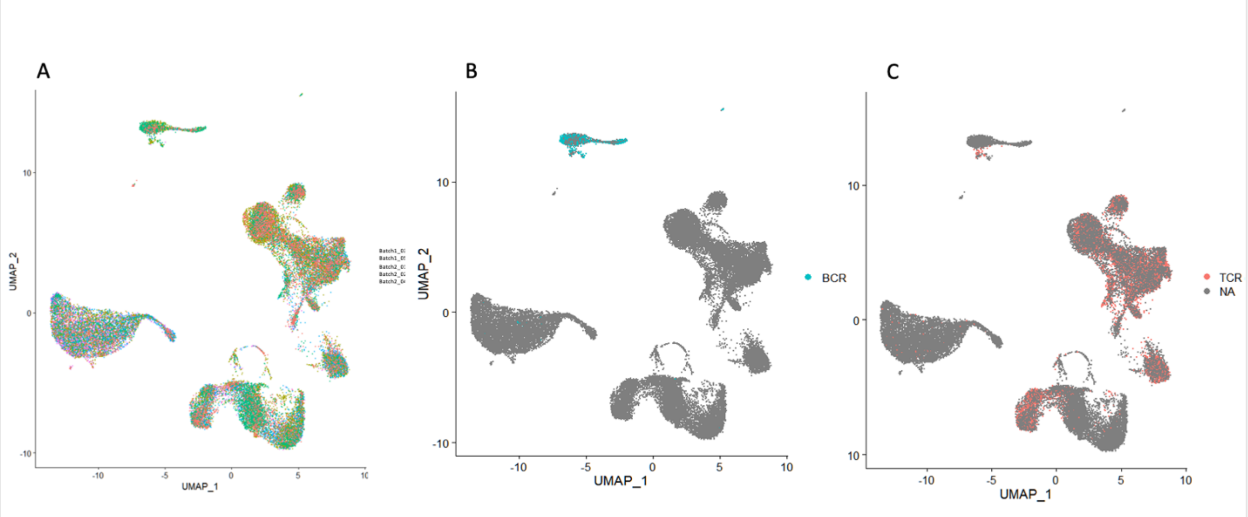

Suppl. Figure 1

UMAP plot after normalization and integration, colored by individual samples. Each dot indicates one cell, with total of 32,848 cells. Well-mixed clusters showed cells were clustered without impact of batch effects. B, C) UMAP showed V(D)J B-cell receptors (B) and T-cell receptors (C) with background of GEX clustering UMAP.

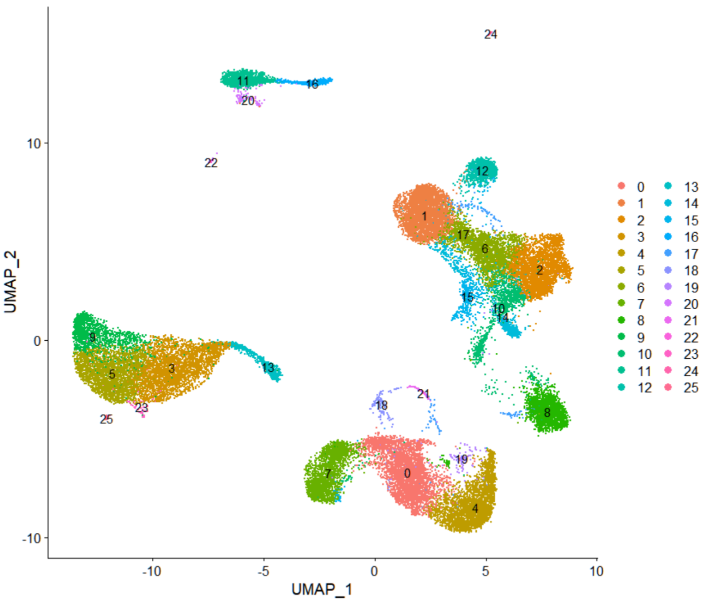

Suppl. Figure 2
UMAP plot of integrated scRNA-seq dataset colored by cell clusters.

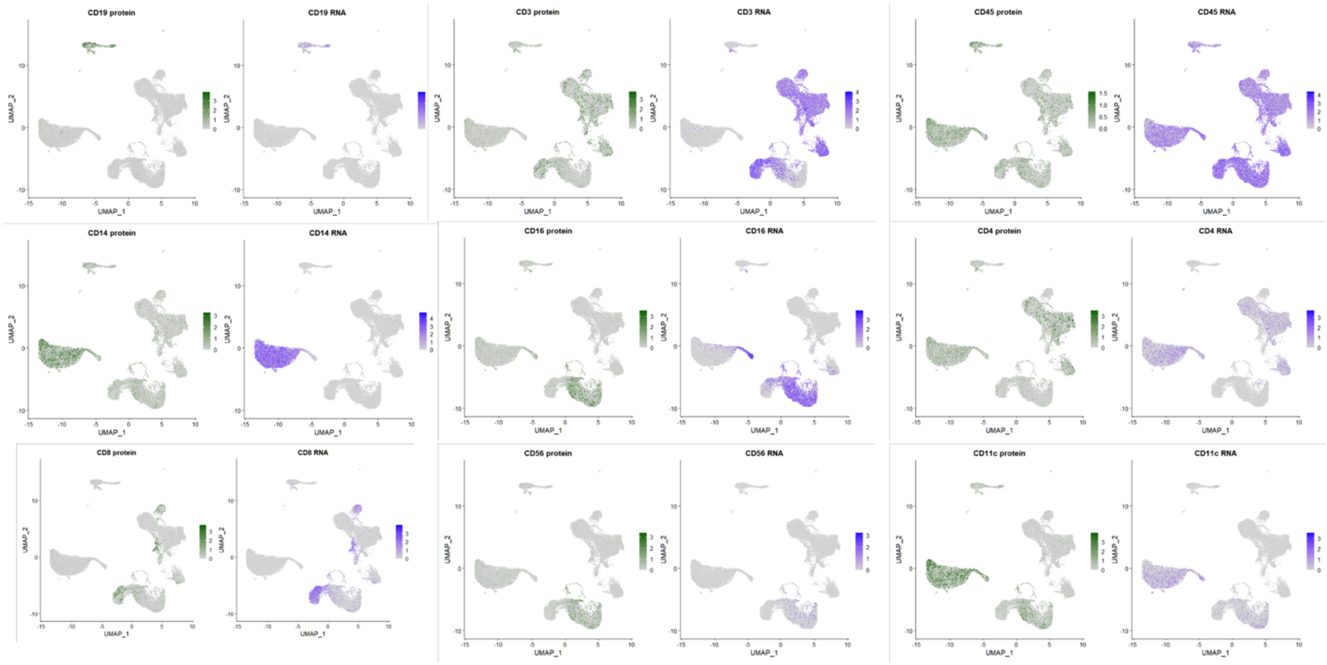

Suppl. Figure 3

Panel of 9 cell surface protein (CSP, left) and their associated RNA expression (right) side by side view: CD19, CD3, CD45, CD14, CD16, CD4, CD8, CD56, and CD11c. Color indicates the level of expression.

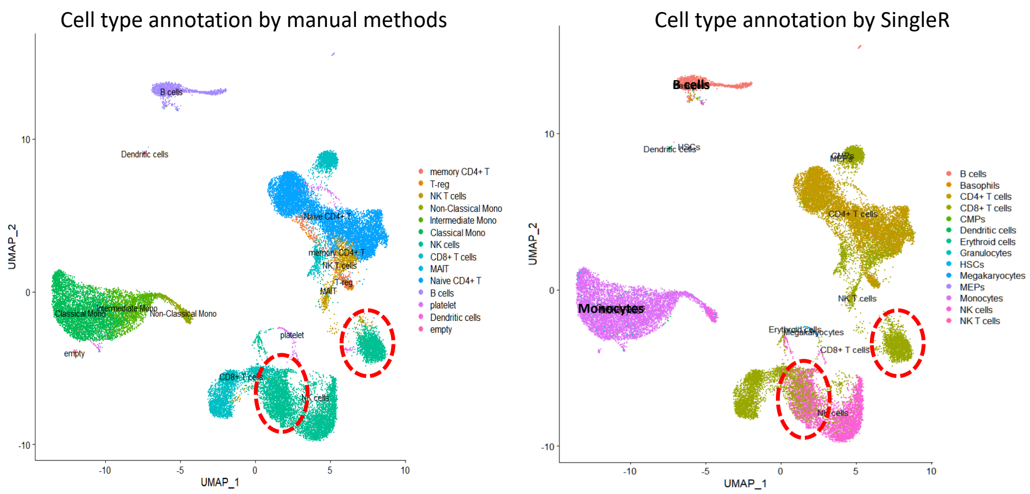

Suppl. Figure 4
Cell type annotation using manual method (e.g., canonical GEX and CSP markers, left) and SingleR (right). 2 red circles indicate the inconsistent annotations.

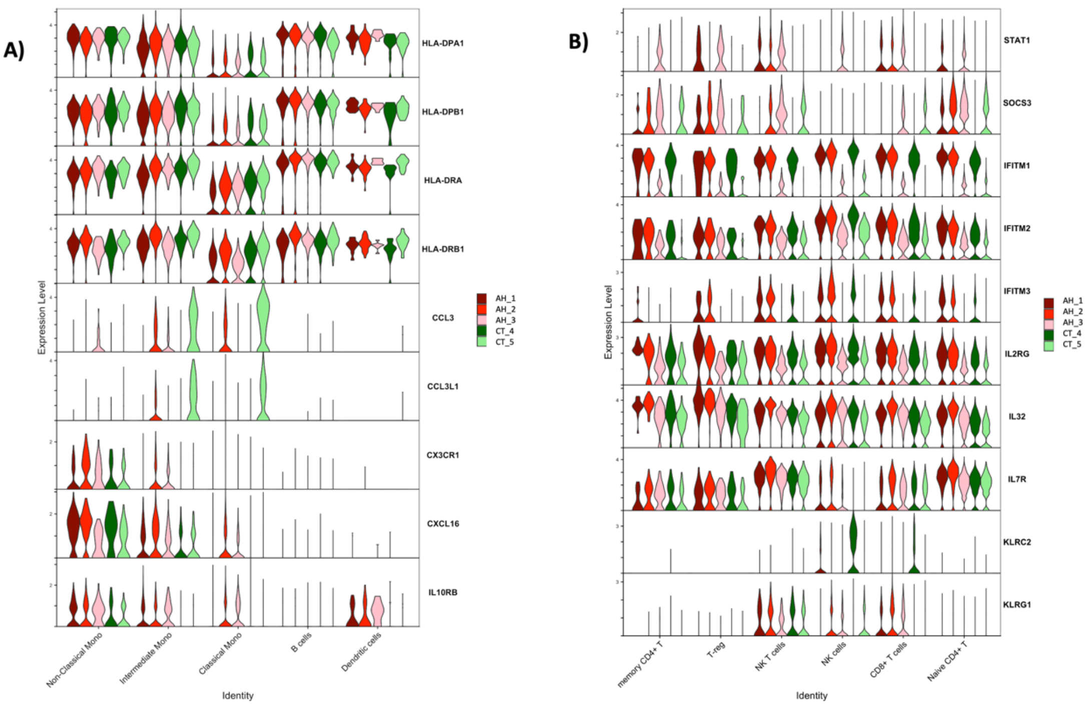

Suppl. Figure 5

Expression levels of select genes across the individual samples from A) monocytes, B cells and dendritic cells; and B) T cells, NKT cells and NK cells shown as violin plot. Red represents AH samples and green represents healthy control.

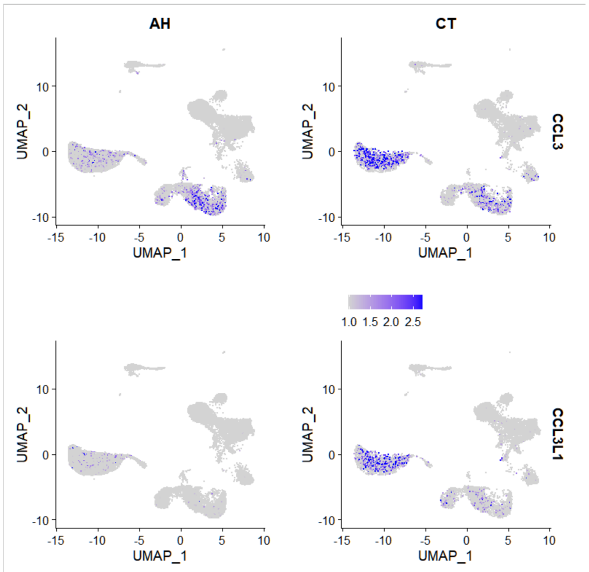

Suppl. Figure 6
Feature plot of CCL3 and CCL3L1 from AH (left panel) and CT (right panel). Each dot represents one cell, and gradient color indicates expression level.

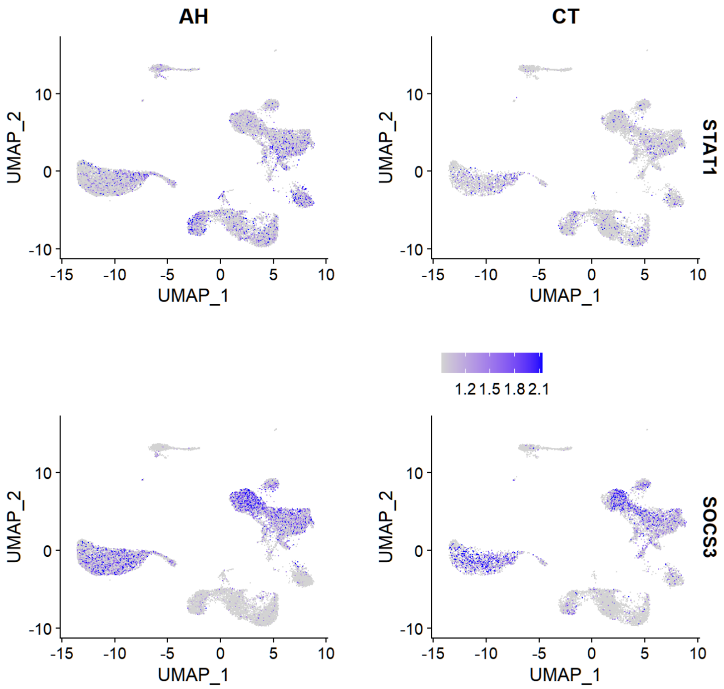

Suppl. Figure 7
Feature plot of STAT1 and SOCS3 from AH (left panel) and CT (right panel). Each dot represents one cell, and gradient color indicates expression level.

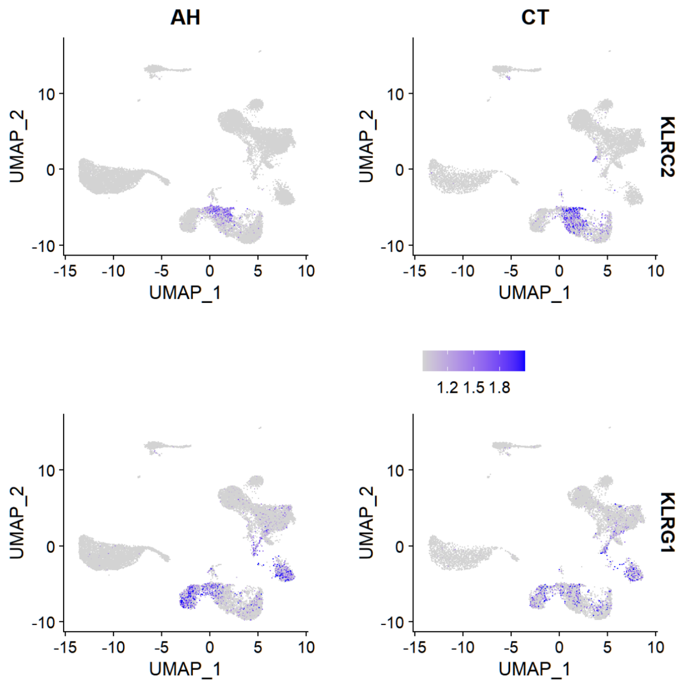

Suppl. Figure 8
Feature plot of KLRG1 (ITIM-Containing Receptor) and KLRC2 (activating NK-cell receptor) from AH (left panel) and CT (right panel). Each dot represents one cell, and gradient color indicates expression level.

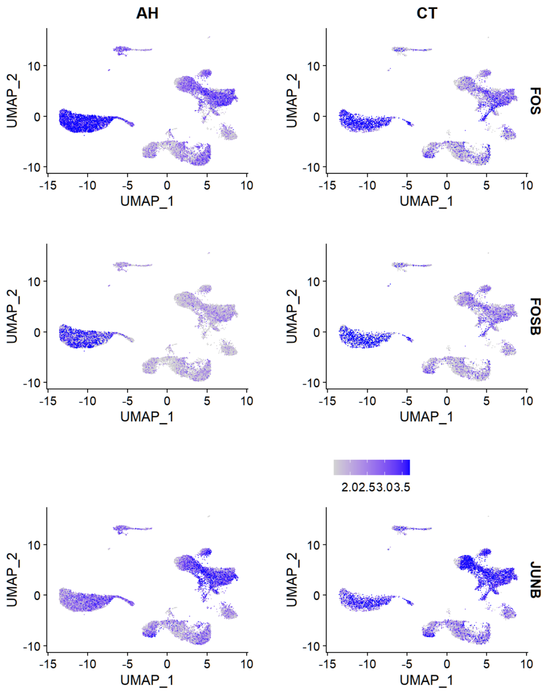

Suppl. Figure 9

Feature plot of FOS, FOSB and JUNB from AH (left panel) and CT (right panel). Each dot represents one cell, and gradient color indicates expression level.

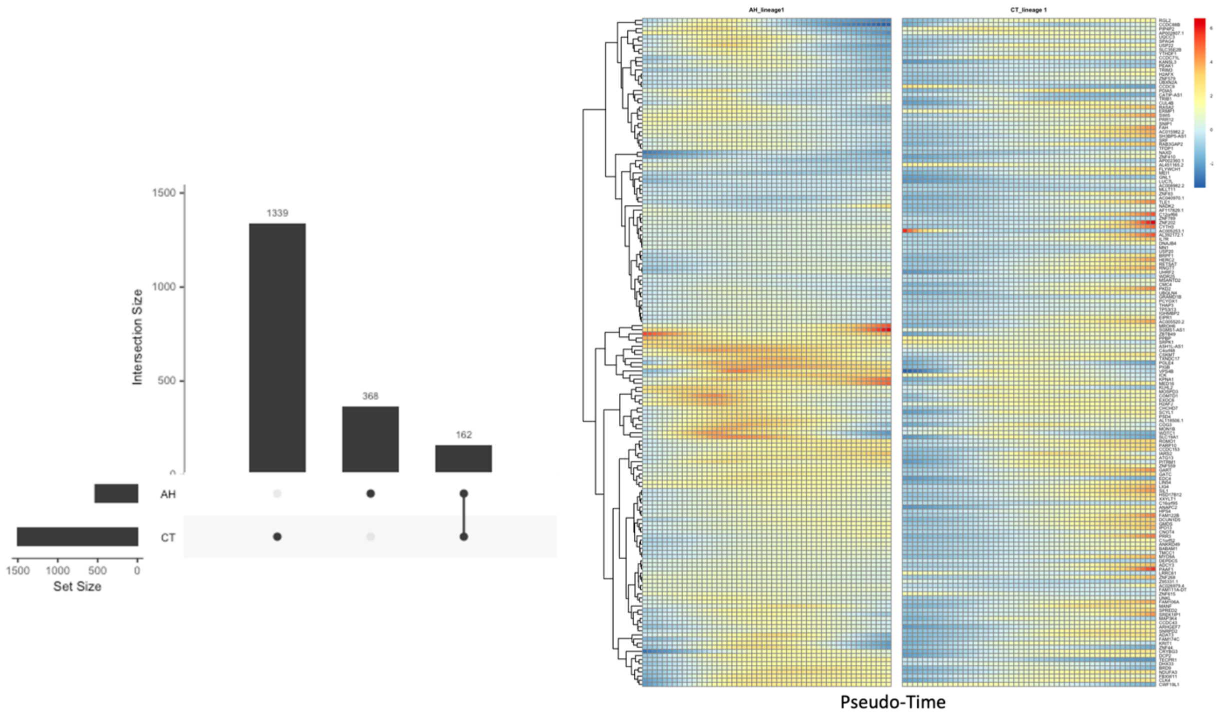

Suppl. Figure 10

A) Histogram of differentially expressed gene counts from monocytes using pseudotime analysis by condition. B) Heatmap of expression levels of 162 genes (overlapped genes as shown on the left) that expressed differently across pseudotime and across conditions by lineage 1 (full lineage, goes from CM (C2, C3, C0) to IM(C1) then to NCM(C4)). X-axis represents pseudotime from left to right.

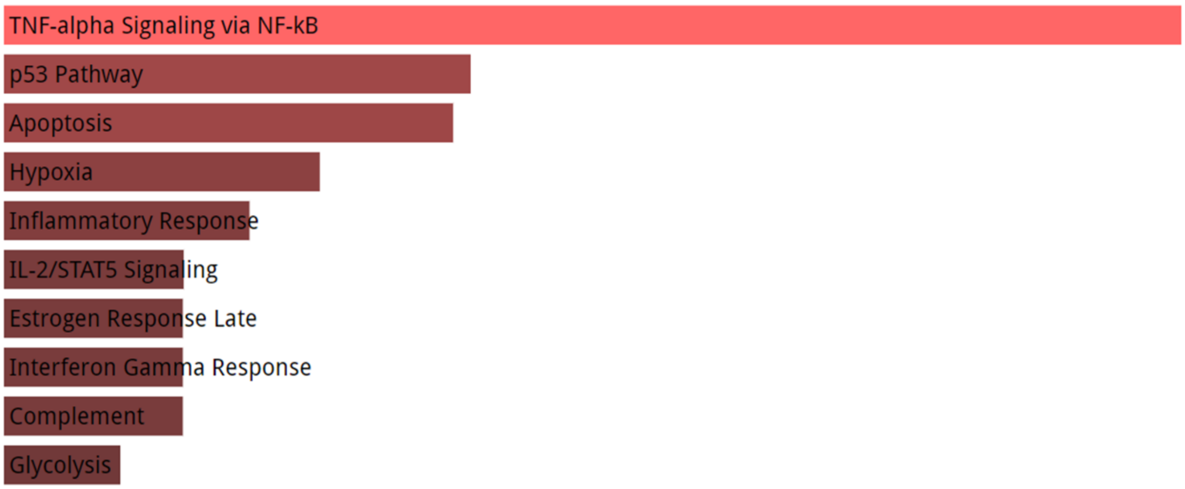

Suppl. Figure 11

Barplot of MSigDB hallmark pathway enrichment of top 87 genes sorted by p-value ranking.

Red indicates p-values <0.05.

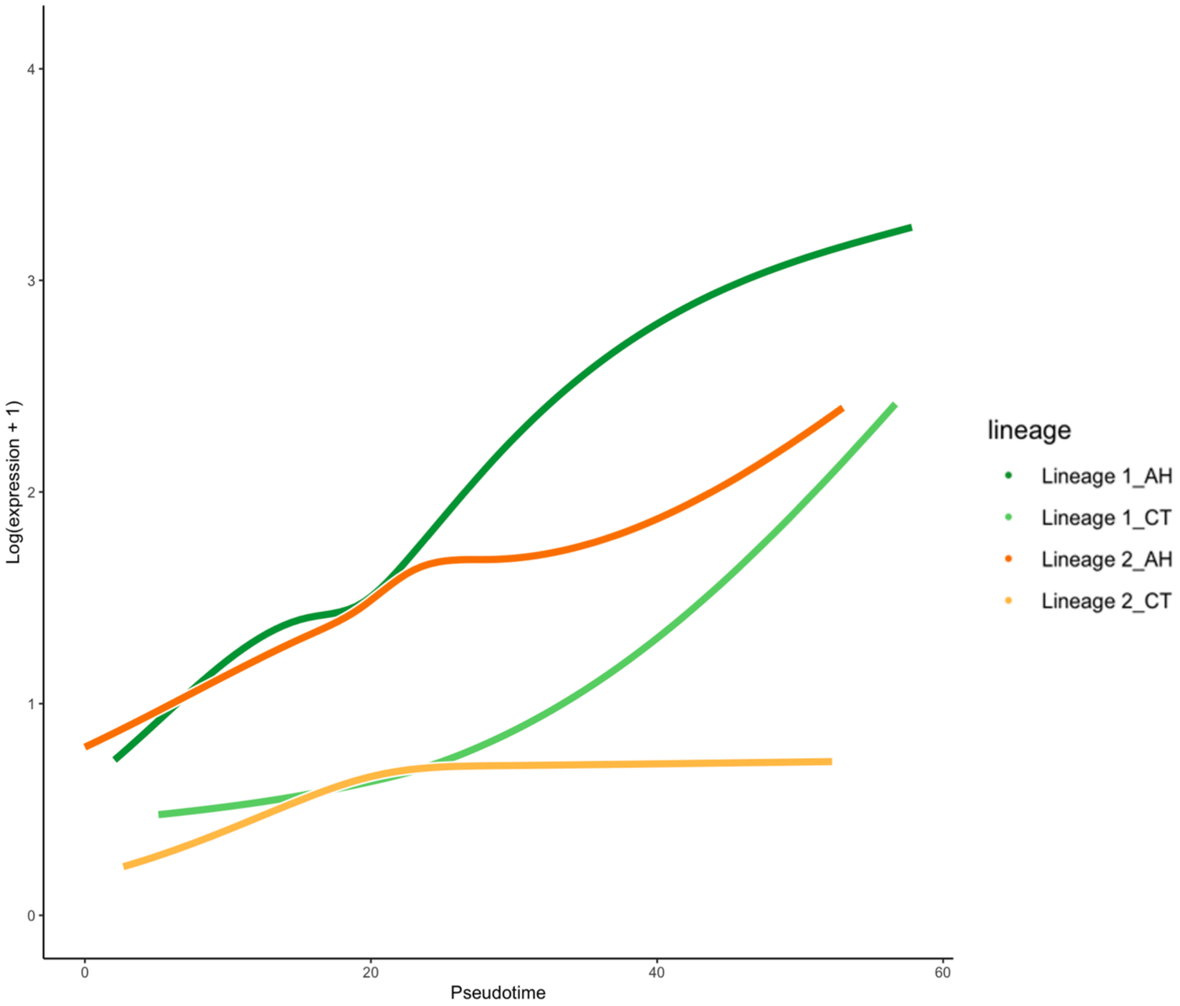

Suppl. Figure 12

Average gene expression levels across pseudotime for each of the lineages from all genes. Green lines are lineage 1, yellow lines are lineage 2. Darker lines are AH samples and lighter lines are healthy control samples.

References

[1] X. Liu *et al.*, "Data Descriptor : Single-cell RNA- seq of cultured human adipose- derived mesenchymal stem cells," *Nat. Publ. Gr.*, pp. 1–8, 2019.

[2] Y. Hao *et al.*, "Integrated analysis of multimodal single-cell data," *Cell*, vol. 184, no. 13, pp. 3573-3587.e29, 2021.

[3] T. Stuart *et al.*, "Comprehensive Integration of Single-Cell Data," *Cell*, vol. 177, no. 7, pp. 1888-1902.e21, 2019.
